# Implementing the theory-based advance care planning ACP+ programme for nursing homes: study protocol for a cluster randomised controlled trial and process evaluation

**DOI:** 10.1186/s12904-019-0505-7

**Published:** 2020-01-08

**Authors:** Joni Gilissen, Lara Pivodic, Annelien Wendrich-van Dael, Chris Gastmans, Robert Vander Stichele, Yvonne Engels, Myrra Vernooij-Dassen, Luc Deliens, Lieve Van den Block

**Affiliations:** 1 End-of-life Care Research Group, Vrije Universiteit Brussel (VUB) & Ghent University, Laarbeeklaan 103, 1090 Brussels, Belgium; 2 Atlantic Fellow for Equity in Brain Health, Global Brain Health Institute (GBHI), UCSF & Trinity College Dublin, San Francisco, CA USA; 3 Department of Family Medicine & Chronic Care, Vrije Universiteit Brussel (VUB) & Ghent University, Laarbeeklaan 103, 1090 Jette, Belgium; 4 Centre for Biomedical Ethics and Law, KU Leuven, Kapucijnenvoer 35 Box 7001, 3000 Leuven, Belgium; 5 Department of Pharmacology, Ghent University, De Pintelaan 185, 9000 Ghent, Belgium; 6 Department of Anaesthesiology, Pain and Palliative Care, Radboud University Medical Centre, Nijmegen, The Netherlands; 7 IQ Healthcare, Radboud Institute for Health Sciences, Radboud University Medical Centre, Nijmegen, The Netherlands; 8 Department of Public Health and Primary Care, Ghent University, De Pintelaan 185, 9000 Ghent, Belgium; 9 Department of Family Medicine and Chronic Care, Vrije Universiteit Brussel (VUB), Laarbeeklaan 103, 1090 Brussels, Belgium

**Keywords:** Advance care planning, Complex intervention, Nursing home, Process evaluation, Effectiveness, Care staff, Educational intervention, Implementation

## Abstract

**Background:**

Research has highlighted the need for improving the implementation of advance care planning (ACP) in nursing homes. We developed a theory-based multicomponent ACP intervention (the ACP+ programme) aimed at supporting nursing home staff with the implementation of ACP into routine nursing home care. We describe here the protocol of a cluster randomised controlled trial (RCT) that aims to evaluate the effects of ACP+ on nursing home staff and volunteer level outcomes and its underlying processes of change.

**Methods:**

We will conduct a cluster RCT in Flanders, Belgium. Fourteen eligible nursing homes will be pair-matched and one from each pair will be randomised to either continue care and education as usual or to receive the ACP+ programme (a multicomponent programme which is delivered stepwise over an eight-month period with the help of an external trainer). Primary outcomes are: nursing home care staff’s knowledge of, and self-efficacy regarding ACP. Secondary outcomes are: 1) nursing home care staff’s attitudes towards ACP and ACP practices; 2) support staff’s and volunteer’s ACP practices and 3) support staff’s and volunteers’ self-efficacy. Measurements will be performed at baseline and eight months post-measurement, using structured self-reported questionnaires. A process evaluation will accompany the outcome evaluation in the intervention group, with measurements throughout and post-intervention to assess implementation, mechanisms of impact and context and will be carried out using a mixed-methods design.

**Discussion:**

There is little high-quality evidence regarding the effectiveness and underlying processes of change of ACP in nursing homes. This combined outcome and process evaluation of the ACP+ programme aims to contribute to building the necessary evidence to improve ACP and its uptake for nursing home residents and their family.

**Trial registration:**

The study is registered at ClinicalTrials.gov (no. NCT03521206). Registration date: May 10, 2018. Inclusion of nursing homes started March, 2018. Hence, the trial was retrospectively registered but before end of data collection and analyses.

## Background

Timely advance care planning (ACP) is advocated as an important part of routine nursing home practice. A recent consensus definition defined ACP as a process that supports adults at any age or stage of health in understanding and sharing their personal values, life goals and preferences regarding future (medical) care, including end-of-life care [1]. If a person wishes, the contents of such conversations can be recorded in the form of an advance directive (AD) and may include choosing a durable power of attorney or proxy decision-maker [1, 2].

A number of previous studies in nursing home populations have shown that, if ACP is actually conducted, it can effectively decrease hospitalisation rates and hospital deaths, decrease overall health costs and increase treatment concordant with people’s wishes [3]. However, these findings usually do not come from studies using high-quality methodologies, as was identified in a recent systematic review using GRADE (Grading of Recommendations Assessment, Development, and Evaluation) criteria to assess the quality of the studies that had evaluated effects of ACP in nursing homes. In addition, very few randomised controlled trials (RCT) in this area have been published [3]. Moreover, the uptake of ACP in clinical practice remains limited and nursing home residents’ wishes about their preferred medical treatment and care are often not, or not in time, explored [4–6]. Previous epidemiological studies have shown that uptake is also low in Belgium where only half of deceased nursing home residents had documented wishes or preferences [7] and 38% of residents never engaged in ACP during their two-year stay in a nursing home [6].

Healthcare professionals’ lack of knowledge about ACP and their confidence in conducting ACP, are identified in the literature as prominent factors preventing them from engaging in ACP [8]. Improving this should be a first priority, given that two theoretical frameworks that describe successful ACP specify that sufficient knowledge and self-efficacy are necessary intermediate steps on the pathway to changing outcomes on the patient and family level [9, 10]. To improve the uptake of ACP in regular nursing home practice, we have developed the ACP+ programme for nursing homes in Flanders (Belgium). ACP+ is a theory-based multicomponent intervention focused on helping staff deliver ACP as part of routine nursing home care, implemented in a stepwise manner over the course of eight months with the help of an external trainer. The underlying theoretical model can be found elsewhere [10]. However, the effectiveness of ACP+ and its theoretical assumptions have not yet been tested using a high-quality research design. This article describes the study protocol of a cluster RCT with an embedded process evaluation. The study aims to evaluate the effects of ACP+ on nursing home staff and volunteer level outcomes and its underlying processes of change. The protocol is outlined according to SPIRIT (Standard Protocol Items: Recommendations for Interventional Trials) guidelines [11].

## Methods

### Trial design

**Fig. 1:**
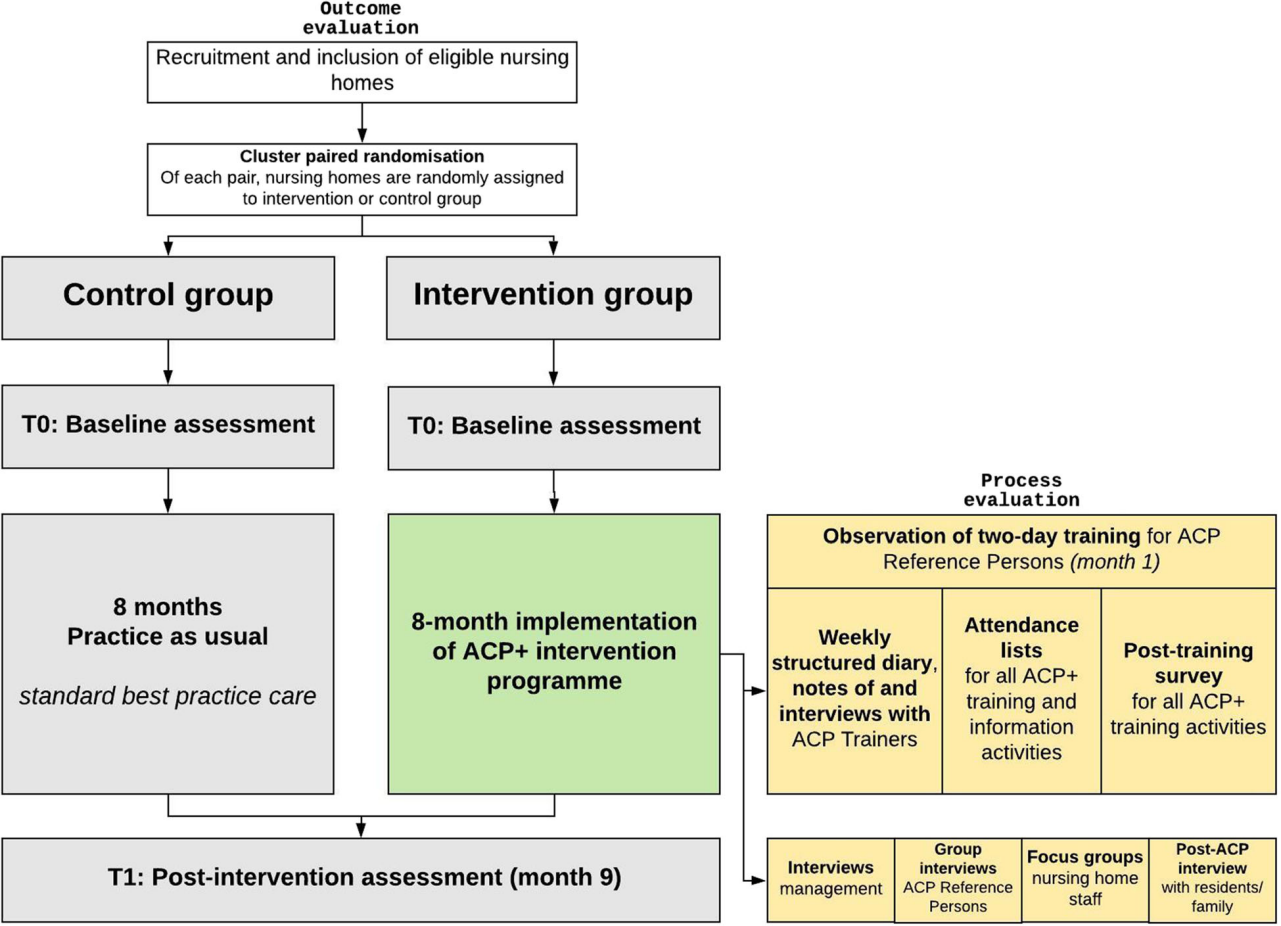
Flow diagram of ACP+ trial. ACP advance care planning; T0 baseline assessment; T1 post-assessment. The yellow blocks indicate the process evaluation data collection methods. The green indicates the intervention group

We will perform a cluster randomised controlled trial (RCT) with embedded process evaluation. It is a superiority trial because it aims to establish whether the intervention is superior to usual practice in effectiveness [12]. The trial will be structured according to a nested cohort pretest-posttest design with a priori matching of clusters [13–15]. Clusters are nursing homes found eligible and willing to participate, which will be matched into pairs (1:1) by (in order) location (province in Flanders, type of facility (public, private non-profit or private for-profit) and number of beds. One of each pair will randomly be assigned to either intervention or control group. A cluster RCT is recommended for this type of study because most intervention components target the entire nursing home. Randomising staff within facilities was not an option as it would have been impossible to prevent contamination among staff on the same wards [16]. Fig. 1 shows the flowchart of the RCT. Immediately after randomisation, baseline outcomes measures are performed (T0) and eight months later, outcome measurements (T1).

The outcome evaluation of the cluster RCT will be accompanied by an embedded process evaluation to evaluate processes of change (i.e. the implementation, mechanisms of impact and context) behind ACP in nursing homes. The design of the process evaluation is informed by the Medical Research Council (MRC) framework for process evaluations of complex interventions by Moore et al. [17] and a previously constructed theory of change [10]. The process evaluation has a mixed-methods design, collecting data throughout implementation of the intervention and post-intervention via structured diaries, notes, attendance lists, observation, post-training surveys and semi-structured interviews and focus groups. The study is registered at ClinicalTrials.gov (no. NCT03521206).

### Participants, intervention and outcomes

#### Setting

The study will be carried out in nursing homes in Flanders, the Dutch-speaking part of Belgium. Nursing homes are included if:

- they have at least 100 beds
- the facility manager expresses explicit motivation to participate in the study and agrees to allocate 0.10 FTE per week for at least two staff members per 30 to 40 nursing home beds to act as ‘ACP Reference Person(s)’.

Nursing homes are excluded if:

- they have taken or are taking part in another research study that is evaluating palliative care services or communication strategies, currently or in the past four years
- they have developed - or are planning to develop during the foreseen duration of the trial - an extensive ACP policy, meaning that (i) all nursing home residents, or their families, regularly receive ACP conversations (two conversations or more each year) or (ii) the nursing home is judged by the researchers as having explicit and detailed ACP guidelines available (corresponding to high-quality ACP procedures and practices).
- major organisational or physical changes to the facility (e.g. building activities or staff re-organisation) are planned or ongoing during the study priod
- they were involved in the development of the intervention programme.

### Study population and respondents

#### Nursing home staff and volunteers

Both the intervention and data collection methods are targeted at multiple staff members and volunteers working in the nursing home. Nursing home staff are people employed by the nursing home and include two groups:

- nursing home ‘care’ staff are nurses, care assistants, psychologists, physiotherapists, occupational therapists, social workers, animators, pastoral or spiritual caregivers, moral consultants, reference persons for dementia or reference persons for palliative care
- nursing home ‘support’ staff are staff working in the nursing home but without having a role in care provision i.e. cleaning, administrative, technical/logistical or kitchen staff who have regular contact with residents or family but do not provide direct care to them.

Nursing home staff are included if they are able to speak and understand Dutch. Volunteers are included if they are registered at the nursing home and able to speak and understand Dutch. Students, interns or volunteers under 18 years old are excluded from participation.

#### Nursing home residents and family

The intervention will not be directly targeted at nursing home residents or family, as ACP+ is a training and support programme directed at nursing home staff level. As part of the process evaluation, we will conduct semi-structured interviews with a small sample of residents and their families from the intervention nursing homes who have participated in ACP conversations. Family members are defined as relatives or friends of the resident and identified by the nursing home care staff. People younger than 18 years, unable to understand or speak Dutch or unable to provide written informed consent are excluded from participation.

### Intervention: the ACP+ programme

The ACP+ programme is a multicomponent theory-based intervention aimed at training and supporting nursing home staff with the implementation of ACP into daily nursing home care and routine practice. It is focused around training and coaching, management buy-in, identifying roles and responsibilities in having ACP conversations with all residents and/or their families, documentation and information transfer, regular follow-up during multidisciplinary meetings and audit, and also includes possibilities of tailoring specific elements to the local context. The programme includes ten intervention components, 22 activities and 17 materials to support its delivery, detailed in Table 4 and Table 1. The components are to be implemented stepwise over the course of eight months, with the help of one or two external trainer(s) whose support decreases as the nursing home becomes more autonomous in organising ACP. These trainers understand that coaching and communication are important to change practice, they have clinical practice experience in nursing homes, experience in delivering palliative care, and in performing ACP conversations. Ultimately, a family physician and a nurse were selected.

**Table 1:** Summary of the ACP+ programme using the Template for intervention description and replication (TIDieR) checklist [18]

TIDieR number*	TIDieR item	ACP+ intervention program
1	BRIEF NAME (name or a phrase that describes the intervention)	The intervention is called “The ACP+ programme” (Dutch: *“Het VZP*^*+*^ *programma”*) and has been developed to implement and improve advance care planning in routine care practice in nursing homes in Flanders, Belgium.
2	WHY (any rationale, theory, or goal of the elements essential to the intervention)	Research shows that only a minority of older people actively engage in ACP, and that there is still a low prevalence of ACP in Flemish nursing homes [6, 19]. Nonetheless, a majority of the growing population of older people would appreciate an opportunity for such discussions and planning [20, 21]. The overall aim of ACP is to improve quality of care, quality of life and quality of dying of residents in nursing homes in Flanders. The theoretical model which was developed in the first phase of intervention development additionally provides a rationale for the individual elements essential to the intervention. This Theory of Change map is reported elsewhere [10].
3	WHAT Materials (any physical or informational materials used in the intervention, including those provided to participants or used in intervention delivery or in training of intervention providers)	17 intervention materials are provided to support delivery of the ACP+ programme: 1. Manual for the ACP Trainer, highlighting key issues of the ACP+ programme and guidance for ACP Trainer to perform his/her tasks 2. Information guide for nursing home management, highlighting key issues and challenges of ACP, explaining ACP+, how it should be implemented, what everyone’s roles are and how they should carry out all the steps within the ACP+ programme 3. Tailoring checklist, including information per intervention activity about the minimum of elements that should be held constant over all nursing homes and which elements can be adapted to each nursing home routines 4. Training manual for two-day training of ACP Reference Persons including educational materials for the ACP Trainer to be used in training 5. ACP manual for ACP Reference Persons including all materials that can be used in the implementation and organization of all intervention activities of the ACP+ program 6. Summary list on which nursing home staff notes all residents and their loved ones, that are eligible for an ACP conversation. This list provides an overview of who scheduled a planned ACP conversation and when. 7. Invitation letter for residents and family, inviting them to participate in information session about ACP 8. ACP information brochure for residents and family 9. Invitation letter for GPs, inviting them to participate in information session about ACP 10. ACP iormation brochure for professionals 11. Training manual for ACP Reference Persons to train other nursing home staff 12. ACP Conversation Guide providing information about initiating and preparing ACP conversations. The guide is structured as follows: A) ideas about a good life and discussing broader views and values (e.g. “What makes your life meaningful?”); B) preferences for current care (“What makes you worry?”), C) the importance of ACP (“Have you ever thought about what kind of care you would want (or not) in case you would be too sick to tell it yourself?”); D) shared care goals (e.g. “Do you feel it is important to make your own decisions with regards to your care? What do you feel is more important: quality of life or living as long as possible, not matter what?”); E) surrogate decision-maker/representative (e.g. in case you were too sick to make your own decisions regarding care, who do you trust to make medical decisions instead?”); F) documenting preferences, including advance directives; G) Place of death; H) other preferences (e.g. “Do you have other wishes that we can take into account?”); I) wishes regarding death (e.g. “Do you prefer to have specific rituals?”); J) revising preferences (e.g. “Under which circumstance would you like to definitely revisit your wishes?”). The conversation guide starts by exploring the broader views of the person and wishes regarding? Current care, and subsequently focuses on future care and end of life and dying. 13. ACP Conversation Tool, a short A4 document that staff can use during ACP conversations. It includes probe questions and brief conversation guidelines following the same structure as the full conversation guide. 14. ACP Document to document outcomes of ACP conversation(s); that can also be used as a transfer document to accompany the resident when transferring between care settings (e.g. ICU). 15. Standardised advance directives 16. Guideline about ACP in dementia for professionals working with people living with dementia [22] 17. ACP audit instrument
4	WHAT Procedures (each of the procedures, activities, and/or processes used in the intervention, including any enabling or support activities)	The ACP+ programme entails 10 intervention components that can be carried out via 22 intervention activities: As part of ‘ACP Trainer’ component (1) Activity 1: Selection and preparation of two ACP (external) Trainers Activity 2: ‘Shadowing’. During the first four months, the trainer follows the selected ACP Reference Persons in their daily job to get familiar with the aspects related to the nursing home, certain routines and ACP-related activities that are already in place As part of ‘Buy-in management’ component (2) Activity 3: Meeting(s) between the ACP Trainer and the nursing home management, representatives of the board of directors, head nurses and the coordinating advisory physician† to explain the project and ask management for their (active) participation (including integrating ACP in the general policy of the nursing home and ensuring staff is able to spend time on their tasks to implement and organize the ACP+ programme and ACP in general, within the routine care). During this meeting they suggest nursing home staff eligible to function as ACP Reference Person‡ (in consultation with staff themselves) Activity 4: Follow-up meetings between management, other decision-makers, ACP Reference Persons and the ACP Trainer. As part of ‘tailoring’ component (3) Activity 5: Tailoring-meeting(s) between ACP Reference Persons, management and important decision-makers‡ about how to fit the implementation of the ACP+ programme to routines As part of ‘ACP Reference Persons’ component (4) Activity 6&7: Two-day interactive training (session 1 and 2) for the ACP Reference Persons Activity 8: Come-back seminar for all ACP Reference Persons As part of ‘information about ACP’ component (5) Activity 9: Information (session(s)) for all residents and their families about ACP in the nursing home during a format that is ‘tailored’ to routines in the specific nursing home setting (e.g. resident/family council, individually, exceptional information session) Activity 10: Information session(s) for all GPs about ACP in the nursing home, including motivating them to consider the wishes and preferences of their patients in (end-of-life) decision-making and to engage in ACP of their patients. GPs are invited to an information session after 5 p.m., accreditation can be arranged. As part of ‘in-house training’ component (6) Activity 11&12: In-house 2-h training sessions (session 1 & 2) to train ‘ACP Conversation Facilitators’ in performing ACP conversations Activity 13: In-house 1,5-h training session to train ‘ACP Antennas’ to educate them how to recognize triggers in residents and family, so they are more willing to have spontaneous ACP conversations according to their competencies and so they know how to pass on information to other staff As part of the ACP planned conversation(s) (7) Activity 14: Exploration of earlier wishes and GP involvement. Activity 15: First planned ACP conversation with resident and family Activity 16: ACP follow-up conversation(s) Activity 17: Documentation of wishes and preferences As part of ‘information transfer’ component (8) Activity 18: Integration of ACP into multidisciplinary meetings so information is shared across professionals in the nursing home As part of ‘coaching’ component (9) Activity 19: One-to-one coaching on request, by ACP Trainer to nursing home staff Activity 20: In-house specialization session 1 (at least 2 hrs): Dementia Activity 21: In-house specialization session 2 (at least 2 hrs): Communication with other healthcare professionals As part of ‘audit’ component (10) Activity 22: ACP audit meeting(s) to discuss ACP procedures with all involved healthcare professionals, the coordinating advisory physician and the management to identify problems and discuss action plans for improvement
5	WHO PROVIDED (intervention provider, their expertise, background and any specific training given)	- ACP Trainers will be available to support nursing homes in implementing ACP into routine care. These trainers are skilled and experienced in change management, have clinical practice experience in nursing homes and in performing ACP conversations. They are able to train other professionals. Their support decreases as nursing homes become more autonomous in organising ACP. - ‘ACP Reference Persons’ are professionals employed by the nursing home who have roles in daily resident care (e.g. head nurses, team coordinators, nurses, palliative care reference persons, reference persons for dementia, psychologists, members of the palliative (support or care) team/working group). The ACP Reference Persons’ main responsibility is to implement and sustain ACP within the nursing home. They market the program, communicate the high priority for nursing home residents, provide education (to ACP Conversation Facilitators and ACP Antennas), conduct ACP conversations with residents and/or family, and perform regular monitoring to audit advance care planning processes, structures and outcomes within the nursing home. - ‘ACP Conversation Facilitators’ or other (head) nurses, palliative care reference persons, reference persons for dementia, psychologists, social workers, care assistants, pastoral or spiritual caregivers, moral consultants and members of the palliative (support or care) team/working group that are willing. These trained conversation facilitators are - together with ACP Reference Persons - responsible for planning and performing regular *manualized* ACP conversations with residents and/or family. - ‘ACP Antennas’ are all others. This is usually staff that do not necessarily provide resident care but do have daily contact with residents and/or family (e.g. care assistants, hair dressers, cleaning staff, administrative staff, volunteers, ...). They will receive a short training in a much easier formulae in recognizing and signalling triggers that can signal the person is ready or willing to engage in ACP.
6	HOW (modes of delivery)	All intervention activities are provided face-to-face, individually, in duo or in groups with a maximum of 15 participants.
7	WHERE (the type(s) of location(s) where the intervention occurred, including any necessary infrastructure or relevant features)	The intervention is meant to improve ACP in nursing homes in Flanders (Belgium). These nursing homes are skilled nursing care facilities where older adults reside who have problems with activities of daily living and/or physical and cognitive functioning [23]. Most residents are still supervised by their GP but since 2000, each nursing home is legally obliged to have a coordinating advisory physician, a GP, preferably trained in gerontology whose tasks include among others, consultancy and conflict mediation in palliative care situations. In addition, nursing homes must cooperate with the geriatric service of the regional hospital and a specialized service of palliative care [24]. The two-day training for the ACP Reference Persons is organised across all nursing homes in a geographically central location. The other training and information sessions are organised in-house. ACP conversations or meetings can be held in a private room in the nursing home.
8	WHEN and HOW MUCH (the number of times the intervention was delivered and over what period of time including the number of sessions, their schedule, and their duration, intensity or dose)	ACP+ should be implemented over the course of 8 months and includes a thorough preparatory or training phase (month 1 to 4) and a follow-up phase (month (5 to 8). Information and training sessions vary from 1 h to two days, depending on the type. ACP conversations are known to vary between 60 and 240 min [25].
9	TAILORING (if the intervention was planned to be personalized, titrated or adapted, then describe what, why, when, and how)	To maximize the fit between individual nursing home needs and ACP+, participating nursing homes have the opportunity, in consultation with the trainer, to choose how they operationalize some activities (e.g. how to fit intervention activities into existing work schedules (e.g. training during lunch, information session for GPs in the evening), how activities are routinely discussed (formally and informally, e.g. through posters, meetings, family council), who needs to be involved in decision-making and how proposed materials can be entered into existing electronic systems).

*ACP* advance care planning; GP general practitioner

*TIDIER items ‘modifications’ and ‘how well the intervention was implemented’ cannot be reported here and can only be described after the study is complete

†Nursing homes are legally obliged to have at least one coordinating and advisory physician (remunerated according to the number of beds), who coordinates medical care in the facility, as well as reference nurses for palliative care [26]

‡Decision-makers are considered to be: head of nursing staff, head of residents’ care, nursing home management. All those involved with decision-making tasks in the nursing home

A key aspect of the programme is the imparting of different roles in the nursing home: ‘ACP Reference Persons’ will be responsible for implementing ongoing ACP within the nursing home; ‘ACP Conversation Facilitators’ work with ACP Reference Persons and are responsible for planning and performing regular ACP conversations with residents and/or family; all other staff, including support staff (administrative, technical, cleaning staff) and volunteers, are ‘ACP Antennas’, who recognise and signal triggers that indicate a persons’ readiness, need or willingness to engage in ACP.

To develop the ACP+ programme, we first applied a Theory of Change approach to develop a theoretical model of all intermediate steps necessary to achieve desired long-term outcomes for nursing home residents and their families [10]. We constructed this model through 1) context analysis of facilitators/barriers that enhance or inhibit ACP, 2) systematic review of preconditions for ACP in nursing homes [8] and 3) two workshops with stakeholders to identify how ACP is expected to work in the local context in order to achieve its desired long-term outcomes [10]. We then operationalised key intervention components – identified as part of this theoretical model – into specific activities and materials, through expert discussions and review of existing ACP programmes, and we evaluated the programme (including the activities and materials) for perceived feasibility and acceptability of its implementation in nursing homes through interviews with nursing home management and staff of five nursing homes, and expert revisions; ethics approval was granted by the Ethics Committee of University Hospital Brussels (2017/31, (B.U.N. 143,201,732,133). A standardised description of the final ACP+ programme, according to the TIDieR checklist can be found in Table 1.

### Control group

In nursing homes that are randomized to the control condition, care will be provided as usual. In case nursing home staff in this group receives training regarding ACP and/or or initiate ACP with residents or families, these nursing homes will remain in the control group. We will perform baseline and follow-up measurement of primary and secondary outcomes in this group, but no process evaluation assessments as the intervention is not delivered there. After the study ends, the control nursing homes will have the possibility of discussing the results of the study with the research team, have access to all intervention materials and receive a one-day training from the external trainers.

### Outcomes

#### Primary outcome

The two primary outcomes are: 1) nursing home care staff’s knowledge of ACP and 2) nursing home care staff’s confidence in their own skills regarding ACP (self-efficacy). These outcomes are measured at baseline (T0) and after eight months (T1). We assess knowledge and self-efficacy as these are identified as necessary intermediate steps for healthcare professionals to be able to actually engage in ACP, according to both social cognitive theory and literature about successful ACP [9, 10, 27].

#### Secondary outcomes

The following secondary outcomes are measured at baseline (T0) and after eight months (T1): 1) nursing home care staff’s attitudes towards ACP and ACP practices; 2) support staff’s and volunteers’ ACP practices; and 3) support staff’s and volunteers’ self-efficacy. Outcomes on support staff- and volunteer level were added because an important part of the ACP intervention is targeting these professional roles. The outcome measure was adapted to this population (See Additional file 1).

#### Outcome measurements

To evaluate ACP knowledge, attitudes, self-efficacy and practices, we developed a questionnaire, based on the questionnaire in a study from Detering et al. [28], which was translated via forward-backward translation and adapted to fit the local context. Items were added based on the Questionnaire Tool for Registered managers from Ulster University [29] and expertise of the multidisciplinary author group. The adapted version of the questionnaire was tested with six researchers who have clinical practice experience with older patients (three registered nurses, one GP, one psychologist and a nursing home volunteer), and through an online survey with 107 healthcare professionals and volunteers active in the Flemish nursing home setting. All items were reviewed and discussed within the author group and questions related to legal issues were additionally reviewed by an expert in Medical Law. Results of this trial will be based on the final version of the questionnaire (Additional file 1).

In the knowledge section of the final version of this questionnaire, respondents are asked to respond to 11 statements (e.g. ‘a nursing home resident can only assign a family member to be his/her legal representative’) ‘true’, ‘false’ or ‘I don’t know’. The self-efficacy section asks respondents to indicate how confident they feel (10-point Likert scale, ranging from ‘little confidence’ = 0 to ‘a lot of confidence’=10 and ‘not applicable’) regarding 12 items (e.g. ‘how confident do you feel about: initiating ACP conversations?’). In the attitudes section respondents are asked to indicate how strongly they agree or disagree (5-point Likert scale ranging from ‘completely disagree’ = 0 to ‘completely agree’ = 5) with 12 statements (e.g. ‘in most cases nursing homes residents do not know enough about healthcare to construct an advance directive’). The construct ACP practices asks about ACP activities in the past six months (e.g. initiating an ACP conversation, drafting of an advance directive, etc). For support staff and volunteers the ‘self-efficacy’ and ‘ACP practices’ sections are adapted to include three items evaluating ‘self-efficacy’ and two items to evaluate ‘ACP practices’. These items are all based on the main questionnaire. Table 2 provides a full overview of outcomes and measures. Questions and scale metrics of the measures are provided in Additional file 1.

**Table 2:** Outcomes and outcome measures of ACP+ trial

Outcome	Respondent	No. of items	Item example(s)
Knowledge of ACP	Nursing home care staff (primary outcome)	11 items*	“A resident can only assign a family member as his/her legal representative”
Self-efficacy towards ACP	Nursing home care staff (primary outcome)	12 items†	“Point out how much confidence you have in your own skills with regard to the following activities/roles: To explain the role of a legal representative to residents and family”
	Support staff	3 items†	“Point out how much confidence you have in your own skills with regard to the following activities/roles: To talk about wishes regarding future care with family members and residents”
	Volunteer	3 items†	Same as above
Attitudes towards ACP	Nursing home care staff	12 items‡	“GPs should be involved actively to help residents draft an advance directive”
ACP practices	Nursing home care staff	8 items	“Did you start an ACP conversation the past six months?”
	Support staff	2 items	E.g. 1: “In the past six months, did you talk with a resident about the next themes: future care and his/her related wishes, dying and death, advance directives?” E.g. 2: “In the past six months, did you talk with a family member or next-of-kin of a residents, about the next themes: future care and his/her related wishes, dying and death, advance directives?”
	Volunteer	2 items	Same as above
Demographic and background information	Nursing home care staff		Age, gender, date of today, number of years working experience in direct patient care, number of years employment in nursing home sector, current function in the facility, highest education, number of hours working in the nursing home per week, whether or not they received training in palliative care or ACP, average number of residents for which they care on regular working day.
	Support staff		Age, gender, date of today, number of years working experience in direct patient care, number of years employment in nursing home sector, current function in the facility, highest education, number of hours working in the nursing home per week, whether they received training regarding one of the following themes: vision and values of the nursing home, palliative care, communication skills, information transfer about resident to other care staff, ACP, other; if they had a personal conversation with a resident that has dementia or Alzheimer’s.
	Volunteer	7 items	Age, gender, date of today, employment status, highest education, number of years active as volunteer, number of years active as volunteer in this nursing home
Structural facility-level characteristics	Key contact person in nursing home	21 items	Type of facility, number of beds recognized by government, number of beds available, number of beds occupied, number of residents per KATZ scale category, umbrella organisation, with which electronic resident file system they work (e.g. GERACC, Care Solutions or others), number of residents died over past six months, average time of stay, availability of specific written guidelines available about palliative care or ACP, availability of patient-specific forms regarding ACP, % of residents died in nursing home, % of residents that has an up-to-date plan regarding end-of-life care, number of residents with written AD, regular multidisciplinary team meetings, number of staff: FTE and heads, number of volunteers registered in nursing home, number of hours per week the coordinating advisory physician is present in facility, number of GPs involved with patients in nursing home

*ACP* advance care planning; *GP* general practitioner; *FTE* full-time equivalent; *AD* advance directive; *KATZ* index of independence in activities in daily living; *GERACC* software package for nursing homes in Belgium

*Response categories: ‘True’, ‘False’ or ‘I don’t know’

†Response categories: 10-point Likert scale, ranging from ‘little confidence’ (=0) to ‘a lot of confidence’ (=10) and ‘not applicable’

‡Response categories: 5-point Likert scale ranging from ‘Completely disagree’ (=0) to ‘Completely agree’ (=5)

§Response categories: ‘Yes’ or ‘No’

#### Other measures

We additionally measure several structural facility-level characteristics of participating nursing homes, and demographic and background information in all participating staff and volunteers. These characteristics are described in Table 2.

### Process evaluation

Via an in-depth process evaluation in the intervention group we will assess:

- *implementation*: defined as the process through which interventions are delivered, and what is delivered in practice [17]. Outcomes involve: how delivery is achieved and what is delivered (dose, reach, fidelity, adaptations).
- *mechanisms of impact*: the intermediate mechanisms through which intervention activities produce intended (or unintended) effects [17]. This involves: responses and interactions from participants with the mediators that might explain changes in outcomes and unanticipated pathways or consequences.
- *context*: factors external to the intervention that may influence its implementation or whether mechanisms of impact act as intended, including outcomes such as contextual moderators (barriers and facilitators) and participant’s intention for maintenance [17].

The process evaluation has a mixed-methods design combining quantitative and qualitative research methods, collected regularly before, throughout and after the intervention period. The results of this process evaluation will enable us to strengthen the links in the theoretical model we have developed in a previous phase [10]. An overview of the process evaluation outcomes (implementation, mechanisms of impact, context) and data collection methods can be found in Table 3.

**Table 3:** Process evaluation methods based on UK MRC guidance on process evaluations of complex interventions (Moore et al. 2012)

Dimension (definition*)	Subdimension (definition*)	Measurements	Data collection method (qualitative or quantitative; timing)
Implementation** (the process through which interventions are delivered, and what is delivered in practice)	HOW delivery is achieved (implementation process: structures, resources and mechanisms through which delivery is achieved)	- Resources: time spent by trainer on preparation and delivery of intervention - Resources: total trial cost associated with delivery of intervention (printing cost training materials, salary trainers, rent training locations and catering) - Implementation process of all ACP+ activities	- Structured diary filled in by trainers (quantitative; weekly) - Expenses from researchers and trainers (quantitative; continuous) - Semi-structured interviews with trainers (qualitative; every 4 months) - Semi-structured group interviews with ACP reference persons per IF (qualitative; after T1)
	WHAT is delivered (the quantity and quality of what is delivered) 1) Dose (how much intervention is delivered)	- Number and type of intervention activities‡ delivered in each IF	- Structured diary filled in by trainers (quantitative; weekly)
	2) Reach† (the extent to which a target audience comes into contact with the intervention)	- Number of ACP Reference Persons of each IF attending two-day training /total number of staff in each IF - Attendance rate of staff during in-house training sessions (for ACP Conversation Facilitators and ACP Antennas) in each IF/ total number of staff in each IF - Number of residents informed about ACP in each IF/total number of residents at T0 in each IF - Number of residents for whom a family member is informed about ACP in each IF/total number of residents at T0 in each IF - Number of GPs informed about ACP in each IF/total number of GPs at T0 in each IF - Number of volunteers informed in each IF/total number of volunteers at T0 in each IF - Number of residents or family members of residents offered minimum one ACP conversation/total number of residents at T0 in each IF - Number of residents with an advance directive/total number of residents at T0 in each IF	- Attendance lists (quantitative; before start of each training or information session) - Survey about number of residents, family and volunteers informed, to be filled in by key contact person in IF (quantitative; after month 6 and at the end) - Information provided by key contact person in IF, based on ACP+ registry document (quantitative; continuous) - Facility level data (quantitative; T1)
	3) Fidelity (the consistency of what is implemented with the planned intervention)	- Number of activities delivered as intended (dose delivered as intended) in each IF/total number of activities - Type of activities delivered, according to participating staff - Content and quality of training workshops for ACP Reference Persons delivered as intended, as observed by researchers - Number of ACP Reference Persons per IF that attended training session scored high on fidelity/total number of care staff at T0 in each IF - Median score of trainer competencies for each training (across and in each IF) - Median score of quality of each training (across and in each IF)	- Structured diary filled in by trainers (weekly) - Semi-structured interview with trainers (qualitative; every 4 months) - Two post-intervention focus group with trained staff across IF (qualitative; after T1) - Semi-structured group interview with ACP Reference Persons in each IF (qualitative; after T1) - Observation of two-day training for ACP Reference Persons by researchers, using checklist of minimum requirements and overall rating of fidelity and quality (quantitative) - Attendance list (quantitative; at each ACP+ training) - Post training survey for participants (quantitative; after each ACP+ training)
	4) Adaptations (alterations made to an intervention in order to achieve better contextual fit)	- Adaptations made to activities of the ACP+ activities (e.g. number, duration, content), according to trainers and Trial Monitor - Experiences with of participants regarding adaptations made and the contextual fit of activities of the ACP+ programme	- Semi-structured interview trainers (qualitative; every 4 months) - Semi-structured group interview with ACP Reference Persons in each IF (qualitative; after T1) - Notes made by Trial Monitor based on communication with trainers and IFs
Mechanisms of impact (the intermediate mechanisms through which intervention activities produce intended (or unintended) effects)	Responses and interactions (how participants interact with the intervention)	- Staff experiences with and views with regard to the ACP+ intervention and activities	- Semi-structured interview with one manager per IF (qualitative; after T1) - Two post-intervention focus group with trained staff across IF (qualitative; after T1) - Semi-structured group interview with ACP Reference Persons in each IF (qualitative; after T1)
	Mediators (intermediate processes which explain subsequent changes in outcomes)	- Evaluation of perceived mediators (or preconditions 1, 2, 6, 7 and interventions 1, 2, 3A, 3B, 4A, 4B, 4C, 6A, 6B, 8 in Theory of Change map [10]), as intermediate processes that might explain changes in outcomes.	- Semi-structured interview with one manager per IF (qualitative; after T1) - Two post-intervention focus group with trained staff across IF (qualitative; after T1) - Semi-structured group interview with ACP Reference Persons in each IF (qualitative; after T1)
	Unanticipated pathways or consequences†	- Potential unanticipated consequences of the ACP+ programme in residents and/or family, in staff, in GP according to participants	- Semi-structured interview with one manager per IF (qualitative; after T1) - Two post-intervention focus groups with trained staff across IF (qualitative; after T1) - One post-intervention focus group with ACP Reference Persons across Ifs (qualitative; after T1) - Three semi-structured interviews with residents and family in each IFs (qualitative; after T1)
Context (factors external to the intervention which may influence its implementation, or whether its mechanisms of impact act as intended)	Contextual moderators† potentially inhibiting or facilitating the implementation, organisation, sustainability and outcomes of ACP	- Contextual barriers and facilitators for 1) implementation (‘the process through which interventions are delivered, and what is delivered in practice’), according to participants - Contextual barriers and facilitators for 2) sustainability (‘the potential for an intervention to become part of routine practice’), according to participants - Contextual barriers and facilitators for 3) outcomes (knowledge, attitudes, self-efficacy and practice), according to participants	- Semi-structured interview with one manager per IF (qualitative; after T1) - Two post-intervention focus groups with trained staff across IF (qualitative; after T1) - Semi-structured group interview with ACP Reference Persons in each IF (qualitative; after T1) - Semi-structured interviews trainers (qualitative; every 4 months)
	Intention for Maintenance† (extent to which the programme is intended to be part of routine organisational practice and policy)	- Staff’s intention for performing ACP+ activities in the future - Organisational intention for long-term implementation - Participants’ recommendations for improving sustainability	- Semi-structured interview with one manager per IF (qualitative; after T1) - Semi-structured group interview with ACP Reference Persons in each IF (qualitative; after T1)

*ACP* advance care planning; *IF* intervention facility; *GPs* general practitioners

Types of training activities: 1) Two-day training for *ACP* Reference Persons (delivered by *ACP* Trainer), across all intervention nursing homes; 2) Two training sessions of each two hours for *ACP* Conversation Facilitators (delivered by *ACP* Reference Persons, supported by *ACP* Trainer), in-house; 2) One training session of 1,5 h for ACP Antennas (delivered by *ACP* Reference Persons, supported by *ACP* Trainer), in-house

*Definition by the MRC Framework by Moore et al. (2012)

**The term implementation is used within complex intervention literature to describe both post-evaluation scale-up (i.e. the ‘development-evaluation-implementation’ process) and intervention delivery during the evaluation period. Within this document, discussion of implementation relates primarily to the second of these definitions (i.e. the quality and quantity of what is actually delivered during the evaluation)

†Added by the research team

**Table 4:**
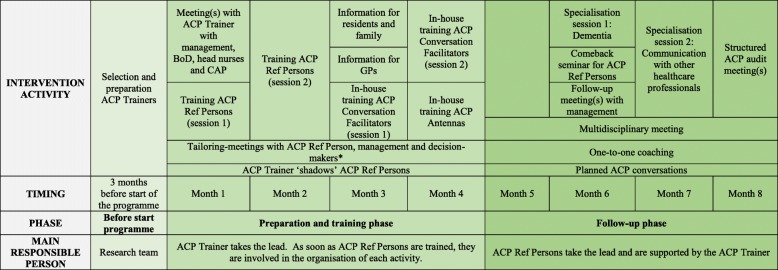
Schematic overview of the ACP + programme. ACP advance care planning; BoD board of directors; CAP coordinating advisory physician; ACP Ref Person advance care planning reference person; GP general practitioner. *Decision-makers are head of nursing staff, head of residents’ care, nursing home management. All those involved with decision-making tasks in the nursing home.

### Sample size

When we assume unequal cluster sizes with a coefficient of variation of 0.17 and mean size of 30 and an intra-cluster correlation coefficient of 0.036 [30, 31], the design effect for a completely randomised cluster randomised trial is estimated at 2.07, and a sample of 161 staff members for each group across 6 clusters will achieve 80.27% power to detect an effect size of 0.5 at a significance level of 2.5%. This number has been increased to 242 staff members per group (total sample size of 484) to allow for an initial response rate of 70% and a staff turn-over of 10%. Current sample size calculation is valid for a completely randomised RCT (hence assuming a matching correlation of zero and assuming the intervention effect is constant across pairs). To compensate for the loss of degrees of freedom introduced by matching, it is suggested to add two clusters per arm [15].

### Recruitment

Umbrella organisations in the nursing home sector in Flanders will be asked to distribute a short informational form about the project and inclusion criteria among their members. If nursing homes express their interest, the researchers (JG and AWvD) will contact them by telephone to introduce the research, do a first check of eligibility, and plan a face-to-face introductory meeting on site. During this meeting, the researchers will inform facility management and head nurse(s) about the study procedures and formally evaluate all inclusion and exclusion criteria. Within two weeks the nursing home’s management will be asked to confirm agreement to participate by signing an agreement form prior to randomisation. In case a facility manager declines to participate, another one fulfilling the eligibility criteria will be selected until a sufficient number of nursing homes are recruited. If this recruitment strategy delivers insufficient nursing homes, the researchers will randomly call a sample of eligible nursing homes from the list of formally acknowledged nursing homes by the national health insurance administration (National Institute for Health and Disability Insurance, in Dutch: Rijksinstituut voor Ziekte- en Invaliditeitsverzekering - RIZIV, in French: Institut National d’Assurance Maladie-Invalidité - INAMI).

### Assignment of interventions

#### Randomisation

After the purposive identification of all nursing homes taking part in the study, they will be matched into pairs (1:1) and one of each pair will then be randomly assigned to the control or intervention group. Facilities that expressed to be interested to participate, are ordered (on a first come first serve basis) on a list which described their region, number of beds and facility type (non-profit, for-profit public/private). We contacted the nursing homes consecutively, starting with the first of the list. After we visited the nursing home, the eligibility assessment was made (using the eligibility criteria). If the nursing home was included, the next on the list was contacted unless there were already sufficient eligible nursing homes in a stratum, in which case the nursing home was skipped and another nursing home with different characteristics was contacted first. Paired randomisation will be performed by an independent and blinded statistician of Vrije Universiteit Brussel (VUB) via computer generated random numbers. The researchers will make a list of all included numbered, including information about facility status (public vs. private without profit objective vs. private with profit objective), location (province within Flanders) and number of beds. The nursing homes will be divided into groups; nursing homes from the same region are grouped. Within each group, nursing homes are subsequently subdivided to match in facility status and then number of beds. The randomisation procedure will be repeated if the numbers of beds are unbalanced i.e. if the difference between the control and intervention groups is greater than 15% of the largest group. Because we will include nursing homes with > 100 beds the difference will not be very great. The randomisation procedure will be repeated a maximum of three times; if an imbalance persists, the last randomisation result will be used for the study. In cases where nursing home staff in the control facilities receive training regarding ACP and/or initiate ACP with residents or families during the study period, these nursing homes will remain part of the control group as this can be part of standard best practice nursing home care. However, to have an extensive ACP policy and practice is an exclusion criterion for nursing homes to be included in the study. This based on the judgement of the two researchers (JG and AWvD), using a list of 12 predefined criteria that define extensive policy and practice. This list is based on a list (of yes/no questions) which is used in a previous Flemish study [32]. Questions range from “The nursing home has a clear and written ACP policy”; “There is oral/written information made available to residents and family regarding ACP, ADs and the assignment of legal representatives” to “Wishes regarding the end-of-life for all nursing home residents (and/or their loved ones) are regularly discussed in team meetings, especially when there are changes”.

#### Blinding (masking)

The nature of the intervention makes it impossible to blind study participants because all those in the intervention group will receive additional training or information. During data collection, the researchers cannot be blinded because they will observe training sessions and conduct interviews with staff as part of the process evaluation, hence will know the staff who work in intervention facilities. The process evaluation will only be conducted in intervention facilities. During data analysis, researchers and statistician will be blinded for the unit of randomisation of each nursing home, using encrypted data.

### Data collection, management and analysis

#### Data collection methods

In each facility, a key contact person (facility manager, head of care, head nurse or quality coordinator) will be identified. After randomisation, this key contact person will fill in the questionnaire concerning the baseline structural facility-level characteristics. In addition, the contact person lists *all* eligible nursing home staff and volunteers. Each eligible staff member/volunteer will be assigned an anonymous code, which will enable the research team to link T0 to T1 data. As part of baseline and post-assessment, they receive a structured self-report questionnaire with his/her personal code. They will put the questionnaire in sealed envelopes and deposit it in a locked letter box (only accessible to the researchers) in a central spot in the nursing home. As was done in a previous Flemish and EU study, two reminders will be sent [19, 32]. Using the anonymous codes, the researchers will register response. For non-responders, the contact person of the nursing homes will be asked to re-distribute the questionnaire to this professional and send out a general reminder. These procedures are repeated eight months after baseline measurement. Newly hired staff and new volunteers are added to the list of codes and will also receive a questionnaire.

Data collection procedures for the process evaluation, described in detail in Table 3, are the following:

- *structured diary of ACP Trainers:* the ACP Trainers keep track of all activities they perform regarding the ACP+ programme by filling in a structured diary on a weekly basis. The diary will be provided by JG via Google Forms, which will be password-protected and stored in a secured folder.
- *notes of ACP Trainers:* after each visit to a nursing home, trainers are asked to write a short report to the Trial Monitor (LP) via e-mail. These reports are held in a secured folder.
- *semi-structured individual interviews with ACP Trainers:* both trainers will be interviewed (60 to 180 min) by one of the researchers at four and eight months. They will be asked for verbal consent to audiotape the interview.
- *attendance lists:* at the beginning of every training or information session an attendance list will circulate among those attending and they will be asked to write down their name and signature. The key contact persons keep the lists in a secure place and will only hand over the total number of participants per session to the researchers.
- *observation of training sessions:* during the two-day training of ACP Reference Persons, the two researchers independently observe the training session using a structured observation checklist.
- *post-training survey:* all staff involved in a training session of the ACP+ programme receive an evaluation questionnaire about the quality of the training and trainer. The surveys will be handed out to the participants at the end of the training by the trainer. Participants will put the survey in a sealed envelope which is then put into the locked box, posted via mail or collected by the key contact person and handed over to one of the researchers. Surveys are anonymised.
- *semi-structured post-ACP interviews with residents and family:* via the key contact person and ACP Reference Persons of each intervention nursing home, at least three residentsand their families are recruited to engage in a semi-structured interview with one of the researchers. After an ACP conversation the staff will ask the resident, family or dyad if they would be willing to participate in an interview. If they respond positively, a date will be planned (preferably shortly after the ACP conversation). At the start of the interview, the researcher will go over the informed consent procedure with the resident and/or their family member. Interviews will last approximately 30 min.
- *semi-structured individual interview with management:* after the intervention, one member of the nursing home management per intervention nursing home will be invited by the researchers for a 30-min interview. The interview will be held in the nursing home and conducted by one of the researchers. Prior to the start of the interview informed consent will be asked and signed.
- *semi-structured group interview with ACP Reference persons:* after the intervention, at least two ACP Reference Persons per intervention nursing home will be invited by the researchers for a 60-min group interview. The interview will be held in the nursing home and conducted by one of the researchers. Prior to the start of the interview informed consent will be requested and signed for.
- *focus groups with nursing home staff:* after the intervention, six to eight staff members per intervention nursing home will be recruited via the key contact person to participate in a 30 to 60-min focus group, held in the nursing home and conducted by one of the researchers. Prior to the start of the interview informed consent will be requested and signed for.

All interviews and focus groups are structured according to a prespecified topic list and audio-taped for analysis purposes. These will all be conducted by JG and AWvD.

#### Data management

Data will be entered as soon as possible after receipt of each questionnaire in a secure open source web-based survey application (Lime Survey). All paper forms, including written informed consent files and questionnaires, are stored in a lockable filing cabinet in a room with restricted access on campus. The participating nursing homes’ names, address and other identifying information will be stored in one file only. This file will be restricted to a few members of the research team (JG, LP, LVDB and AWvD). Consistent with the Good Clinical Practice (GCP) guidelines, the data (without information that is confidential, privacy-sensitive or that could identify individual people) and informed consent files will be stored for 15 years. Other documentation such as potential logbooks of the analyses, published papers, relevant e-mail correspondence etc. will be handed over in digital format to the project lead (LVDB). In case data is shared, a secure method will be used, to ensure it cannot be accessed by anyone outside the research team. This includes email using a suitable encryption programme, with the password sent by another method (usually telephone) or post in a secure envelope.

#### Analysis

##### Outcome analysis

We will calculate summative scale scores for both primary and secondary outcomes. The resulting scale score for an individual is the sum of the individual item scores. For the knowledge items instructions are provided to check correct answers. If people answered, ‘I don’t know’, this will be scored as an incorrect item. The summative scale score of knowledge is sum correct knowledge items of 11 correct/incorrect answers. The summative scale score of ‘self-efficacy’ is the sum of self-efficacy items on a 10-point scale, ranging from 1 to 10, with 12 items. The primary statistical analyses will use an intention-to-treat (ITT) approach. In ITT the outcome data from all of the samples who were enrolled and randomised to the intervention or control group will be accounted for in the main analyses in the original groups to which they were randomised, regardless of whether or not they completed the ACP+ programme. We will fit a linear mixed model with condition, time and time*condition as fixed factors and with a random intercept for nursing home pair, random slope for time, condition and time*condition at the level of nursing home pair, random intercept for member, random slope for time at the level of member. The need for random slopes will be tested by comparing the difference between − 2 log (max) REML likelihoods with a ${\chi}_{1:2}^2$ distribution (using a mixture of chi-square distributions). In case of convergence issues, random slopes will also be left out of the model. Estimated cluster-adjusted means with corresponding 95% CI will be reported at T0 and T1, both for the intervention and control group. Differences in mean change (post- measurements minus baseline) between the intervention group and the control group (interaction group*time) will be calculated. All analyses will be two-tailed and considered significant if α = 0.025. Data will be analysed in SAS, R and IBM SPSS.

##### Analysis of process evaluation data

We will calculate descriptive statistics for quantitative measures (attendance lists, structured diaries, post-training surveys). All qualitative data and transcripts from (group) interviews and focus groups will be analysed using thematic content analysis (via both inductive coding into themes [33] and deductive coding using the theory of change model [10]). The analysis will be carried out by at least two researchers, independently from each other; NVIVO (qualitative data analysis software) will be used for analysis.

### Trial monitoring

The researchers will continuously monitor responses using MS Excel sheets. A Trial Monitor (LP), will be put in place to monitor, together with the research team, the course of the trial. She will act to oversee the progress of the trial and to ensure it will be conducted in accordance with the protocol and GCP [34]. She will also function as main contact person for participating nursing homes to report problems or to ask questions regarding the trial. All data entry will be performed by paid student(s) who are not involved in the research and hired to perform data entry alone. Data will be entered as soon as possible after receipt of each questionnaire in Lime Survey. The Trial Monitor will be responsible for checking and merging trial data. Independent double data entry will be required for 10% of the data to assess accuracy and to avoid data typing or editing errors. We will follow the guidelines of the EMGO’s (Scientific Quality Committee Amsterdam) Quality Handbook regarding data entry accuracy [52]. After data entry, a second database will be created into which a random sample of questionnaires (selected by LP) can be re-entered. The data entry programme identifies double data entry when the second entry is completed correctly. In addition, the researchers (JG or AWvD) will check for and delete duplicate data entries after all data have been entered. If the number of errors on any given questionnaire exceeds 3%, the entire questionnaire must be re-entered. With regard to handling missing data, researchers will register the anonymous code (of eligible participants in the primary outcome measurement) for which no survey was received (MS Excel sheet). These codes will be signalled to the contact person who will be asked to send/present a reminder (i.e. the usual questionnaire). If forms have not been returned, up to two reminders are sent out.

### Ethical considerations

#### Potential Harms

The entire team, including an ethicist involved in the research team (CG), is committed to minimize such risks of harm and maximize the benefits for potential participants. However, this study will carry little to no risk to the participating staff and volunteers. Participating staff and volunteers may feel uncomfortable discussing end-of-life care with residents/family and are only included in the training sessions if they are willing to participate. Sensitive and disturbing questions are avoided in the questionnaires and staff may at any point leave a training session or discontinue completing questionnaires, without stating reasons.

Participation in ACP by residents and their family has been considered highly beneficial with little or no burden associated with participation [3]. They may feel uncomfortable discussing questions about quality of life, or end-of-life care preferences about treatment or envisaging themselves as lacking cognitive capacity. Although sensitive and disturbing questions are avoided in the qualitative interviews, it cannot be fully excluded that some people may feel distressed in the process. Participants are free to withdraw their participation from interviews at any stage, and it will be stressed to staff in the training sessions that ACP should be adapted to the individual, considering his/her readiness and willingness to engage in ACP. ACP in this programme is considered a voluntary process for residents and family to engage in.

A series of procedures will be put in place to identify and handle any sign of distress in residents, relatives and nursing home staff/volunteers (e.g. where the participant contacts the researcher): 1) the contact details of the researchers are mentioned on all documents (including training materials for staff/volunteers and leaflets that can be distributed to residents/family) stating they can contact us in cases of distress; 2) if specific concerns arise, the researcher is advised to direct the participant to resources of help if appropriate (e.g. network for palliative care that is available within each region or a support telephone line for both general public and healthcare professionals; http://leif.be/leiflijn/). If we encounter bad practice in a participating nursing home, we will organise a meeting with the research team, followed by the possibility of an informal complaint to the nursing home management, or a formal complaint if this is deemed necessary. In addition, in the process evaluation, we will monitor unanticipated consequences.

#### Anonymity and confidentiality

We ensure anonymity and confidentiality of all participants throughout the study. The involved researchers will never be informed nor be able to be informed of the participating staff’s and volunteer’s identity, or other personal data that can reveal their identity. In each nursing home, a pseudonymising process will take place. Each eligible staff member/volunteer will be assigned an anonymous code, which will enable the research team to link T0 to T1 data. These lists linking names to codes are held by the contact person in the facilities. To have a spare in case the list gets lost, a duplicate will be kept by the Trial Monitor in a sealed envelope located in a locked space. This envelope can only be opened by the contact person in the facility. To preserve the anonymity of the resident and his/her family, no data will be collected from the administrative or medical files. If they agree to participate in interviews or recordings, their names (and nursing home) will be changed when transcribing the recordings. To protect residents’ and relatives’ privacy during the qualitative interviews, nursing home staff, management and volunteers shall be interviewed separately. When interviews are held, a privacy sign will hang at the door.

## Discussion

There is a lack of high quality trials to evaluate the effectiveness of ACP, especially in nursing homes [3]. This cluster randomised controlled trial (RCT), designed to evaluate the effects of the multicomponent theory-based ACP+ programme in Flanders, is unprecedented and will provide important evidence concerning the effectiveness of ACP on nursing home staff and volunteer level outcomes. With accompanying process evaluation, this project will contribute to providing evidence on the effectiveness of ACP in nursing homes and will enable us to provide insights into how and under what circumstances ACP is implemented in nursing homes and hence to develop better implementation strategies.

This study has several strengths. Firstly, while there are very few high-quality studies that evaluate the effects of ACP in nursing homes, and in particular very few cluster RCTs [3], we contribute to filling this gap by planning and designing this proposed study according to recent recommendations in the conduct of high-quality RCTs [35, 36]. The study design follows that of a previous trial conducted by members of the research team [32]. Therefore, the study protocol has been proved feasible and successful in this study population. Secondly, systematic reviews of ACP highlight that RCTs should be supported by process evaluations that explore implementation issues and identify ‘active elements’ [37] which is an important element of this study. ACP is a complex intervention that ideally targets both organisational and behavioural aspects and is highly influenced by its context (e.g. staffing levels in nursing homes) [17]. Understanding these underlying processes of change can improve our understanding why ACP achieves or fails to achieve intended changes in residents, family or nursing home staff [17]. It can also facilitate the future comparison of similar interventions and the translation to clinical practices or other settings and contexts [17]. We were able to design and plan a process evaluation which is theory-based and structured according to recent guidance [10, 17], enabling us to answer the frequent calls for more transparency in trial results and provide reasons why the intervention did or did not lead to hypothesised effects. As such we will be able to limit something that happened in a recent trial in the Netherlands, where researchers were unable to explain why no effects were found on primary and secondary outcomes [38]. In addition, the results of this process evaluation will enable us to strengthen the links in the theoretical model we have developed in a previous phase [10]. Hence, we will be able to present a theory of how and under what circumstances ACP achieves or fails to achieve desired outcomes. Thirdly, all current trials regarding ACP in nursing homes evaluated outcomes on patient/family level or healthcare use alone (e.g. knowledge of ACP, satisfaction with care, hospitalisation admission rates, number of ADs) [3]. None evaluated the effects of ACP on the level of nursing home staff, while almost all current ACP interventions in nursing homes are educational programmes targeting the knowledge, attitudes or confidence in ACP of professionals [3, 37, 39]. Given that one of the main and most consistently reported factors potentially hindering the completion of ACP is in fact insufficient knowledge of and self-efficacy in ACP among healthcare professionals [8, 40], studying whether and how these educational ACP interventions affect staff outcomes is highly necessary. Considering that a summative evaluation of the effectiveness of our intervention cannot rely on one outcome measure, such as knowledge, we included self-efficacy as primary outcome. Self-efficacy has been identified in social cognitive theory as a mediator for translating knowledge into action (i.e. ACP practices). The results of this trial will be the first to provide evidence of the effects of a complex ACP intervention on staff level outcomes in nursing homes.

The study also has some limitations. Firstly, the most important shortcoming is the limited evaluation of outcomes on resident and family level. For several reasons we chose not to include a primary or secondary outcome for the evaluation of effectiveness of ACP+ at resident or family level. Based on previous research [10], we argue improving quality of care, life and dying is beyond the ceiling of accountability (cf. the point at which we stop accepting responsibility for achieving those outcomes solely through the intervention programme), and the likelihood of finding an effect is limited, as was shown in other trials [38]. Because improving staff level outcomes is a necessary precondition before being able to change outcomes for residents and families, we feel this is an important first step in the effectiveness assessment of ACP+. Follow-up funding will enable us to also assess – retrospectively - whether the ACP+ programme had an effect on care concordance at the end of life, based on chart reviews and family interviews of nursing home residents who died during trial period [41, 42], and we will include residents and their families from intervention nursing homes in the process evaluation to evaluate their experiences. We do stipulate that this rationale underlying the study’s aim, generates an additional study limitation, given that changes in staff knowledge/self-efficacy may lead to changes in both behaviour as well resident outcomes. This is an assumption which might have face validity but is not yet supported by evidence about causal inference. We will also not assess economic outcomes simultaneously, which is recommended by recent reviews of ACP effectiveness in older adults [43]. Secondly, because the recruitment follows convenience sampling, there can be systematic differences between those who choose to participate in the ACP+ trial and those who do not. Thirdly, blinding participants (nursing homes and staff) and researchers will not be possible during the study period. During data analysis however, researchers will be blinded. A recent review which used the Oxford Quality Scale to assess methodological trial quality, showed this has not been possible in any of the past trials [43]. This might affect the answers of nursing home staff/volunteers who know they are in an intervention group. Fourthly, we adapted, developed and preliminarily tested a survey to measure knowledge, self-efficacy, attitudes and practices ourselves. However, the self-efficacy scale from Baughman et al., published in 2016, showed high internal consistency and some evidence of convergent, known groups, and predictive validity in family physicians and might be used in the future for similar research, after being tested in this particular population [44]. In addition, responses of staff and volunteers of intervention groups may be affected by their knowledge of their allocation because blinding will not be possible. Finally, because of the high staff turnover in nursing homes it will be unavoidable that throughout the study period of eight months, some staff will change jobs before follow-up data can be collected [45]. This also means that some nursing home staff will not have the possibility to provide baseline data but will be engaged in providing post-assessment at T1.

## Conclusion

The ACP+ study will be the first cluster randomised controlled trial aimed at evaluating the effectiveness of the multicomponent, theory-based ACP+ programme to support implementation of ACP in nursing homes in Flanders (Belgium). Combined with an in-depth process evaluation, this study will add considerably to the evidence on the implementation of ACP in routine nursing home care. Considering the expected large increase of older adults needing end-of-life care in a nursing home setting, such high-quality trials are urgently needed to provide essential knowledge to improve comparison between ACP programmes and translation into care practices.

## Supplementary information

**Additional file 1.** Items in measures. List of questionnaires used to assess primary and secondary outcomes of the trial.

## Data Availability

The datasets used and/or analysed during the current study are available from the corresponding author on reasonable request.

## Abbreviations

ACP: Advance care planning
AD: Advance directive
FTE: Full-time equivalent
GCP: Good Clinical Practice
GP: General practitioners
ICC: Intra-cluster correlation coefficient
MRC: Medical Research Council
RCT: Randomised controlled trial
