# Supplementary material for: Implementing the theory-based advance care planning ACP+ programme for nursing homes: study protocol for a cluster randomised controlled trial and process evaluation
Source: BMC Palliat Care. 2020 Jan 8;19:5. doi: 10.1186/s12904-019-0505-7 (PMC6950862; doi:10.1186/s12904-019-0505-7)
Supplement: Supplementary file 1 — Additional file 1. Items in measures. List of questionnaires used to assess primary and secondary outcomes of the trial. [file 12904_2019_505_MOESM1_ESM.docx]

**OVERVIEW OF ITEMS IN MEASURES**

**ACP Knowledge**

11 true/false items

Scale metrics: ‘true’ or ‘false’ or ‘I don’t know’

|  | Item included in questionnaire | | | |
| --- | --- | --- | --- | --- |
|  | Care staff | GP | Support staff | Volunteer |
| 1. An AD allows a resident to communicate his will regarding healthcare in case he would lose his/her cognitive capacity in the future *(true)* | X | X |  |  |
| 2. A representative has the power to make decisions regarding healthcare in case the resident is no longer able to do this himself *(true)* | X | X |  |  |
| 3. A resident can only assign a family member as his representative. *(false)* | X | X |  |  |
| 4. A family member can refuse treatments instead of a resident that has no cognitive capacity *(true)* | X | X |  |  |
| 5. A physician is committed to perform all invasive treatments if a resident or family members asks, independent of potential advantages or disadvantages of those treatments *(false)* | X | X |  |  |
| 6. According to the law of Patient Rights both a positive and negative AD is binding *(false)* | X | X |  |  |
| 7. A residents living with dementia can change his/her AD. *(true)* | X | X |  |  |
| 8. Each family member of a resident living with dementia can change this person’s AD *(false)* | X | X |  |  |
| 9. If a resident that has no cognitive capacity (e.g. someone with severe dementia) has not assigned a representative, it is established by law who will take his/her place in decision-making *(true)* | X | X |  |  |
| 10. According to the Law on Euthanasia a physician can perform euthanasia if a person is in an irreversible coma, in case that person has completed a written AD for euthanasia *(true)* | X | X |  |  |
| 11. Residents that have no cognitive incapacity and are not terminally ill, have the right to refuse treatments, even if this decision can lead to death *(true)* | X | X |  |  |

ACP advance care planning; AD advance directive; GP general practitioner

**ACP Self-efficacy**

12 items to which participants indicated self-perceived confidence

Scale metrics: 10-point Likert scale with 1 ‘not at all confident’ and 10 ‘very confident’, including answer category to indicate ‘not applicable’

| Item included in questionnaire | | | |  |
| --- | --- | --- | --- | --- |
| Care staff | GP | Support staff | Volunteer |  |
| 1. Initiating ACP conversations | X | X |  |  |
| 2. Discussing disease and treatment options with a resident within the context of ACP | X | X |  |  |
| 3. Discussing wishes and preferences for future care | X | X | X | X |
| 4. Explain the role of a representative to residents and family | X | X |  |  |
| 5. Respond to questions of residents regarding ADs | X | X |  |  |
| 6. Respond to questions of the family regarding ADs | X | X |  |  |
| 7. Correspond to a residents’ written wishes | X | X |  |  |
| 8. Knowing legislation regarding ADs | X | X | X | X |
| 9. Talking to family members about wishes for future care | X | X |  |  |
| 10. Talking about general issues regarding dying and death | X | X | X | X |
| 11. Conduct a conversation regarding ACP with residents living with dementia | X | X |  |  |
| 12. Conduct a conversation regarding ACP with family members of residents living with dementia | X | X |  |  |

ACP advance care planning; AD advance directive; GP general practitioner

**ACP Attitudes**

12 items to which participants indicate to which degree they agree

Scale metrics: 5-point Likert scale with 1 ‘totally disagree’ and 5 ‘totally agree’

|  | Care staff | GP | Support staff | Volunteer |
| --- | --- | --- | --- | --- |
| 1. In most cases residents know enough about healthcare to complete an AD. | X | X |  |  |
| 1. GPs must be actively involved to help residents to complete an AD. | X | X |  |  |
| 1. GPs are mostly informed about the wishes from their residents with regard to end of life care, without an AD or any other written document of ACP. | X | X |  |  |
| 1. The information in an AD is often sufficient to guide treatment. | X | X |  |  |
| 1. Family is often informed about the resident’s wishes regarding end-of-life care. | X | X |  |  |
| 1. It is emotionally draining to help residents complete an AD. | X | X |  |  |
| 1. An ACP conversation should be held with every resident. | X | X |  |  |
| 1. ACP can facilitate the decision-making regarding the end of life for family members from residents living with dementia. | X | X |  |  |
| 1. Residents living with dementia can lose hope after an ACP conversation. | X | X |  |  |
| 10. For most residents with beginning dementia it is useful to receive information about their disease trajectory and possible options for future care and treatment. | X | X |  |  |
| 11. A resident with dementia should be involved in an ACP conversation. | X | X |  |  |
| 12. During an ACP conversation with a resident living with dementia, a family member should be present. | X | X |  |  |

ACP advance care planning; AD advance directive; GP general practitioner

**ACP Practices**

6 ACP practices to which participants must indicate their involvement in the last 6 months

Scale metrics: ‘yes’ or ‘no’ answer

|  | Included in questionnaire | | | |
| --- | --- | --- | --- | --- |
|  | Care staff | GP | Support staff | Volunteer |
| 1. Started an ACP conversation | X | X |  |  |
| 2. Documented the outcomes of an ACP conversation in a resident’s file | X | X |  |  |
| 3. Completed an AD with a resident | X | X |  |  |
| 4. Made an estimation if someone was capable of completing an AD | X | X |  |  |
| 5. Had an ACP conversation with a resident that has dementia | X | X |  |  |
| 6. Had an ACP conversation with family of a resident that has dementia | X | X |  |  |

ACP advance care planning; AD advance directive; GP general practitioner

**6 additional questions related to ACP practices**

Scale metrics are included in the questions

|  | Care staff | GP | Support staff | Volunteer |
| --- | --- | --- | --- | --- |
| 1. With how much residents have you started an ACP conversation over the past 6 months? *(numerical value)* | X | X |  |  |
| 2. With how many family members have you started an ACP conversation over the past 6 months? *(numerical value)* | X | X |  |  |
| 3. Who generally starts the ACP conversation in your nursing home? *(multiple choice)* | X |  |  |  |
| 4. When is an ACP conversation usually initiated? *(multiple choice)* | X |  |  |  |
| 5. Who usually participates in an ACP conversation in your nursing home? *(multiple choice)*  6. Are ACP conversations documented? If yes, how? *(multiple choice)* | X |  |  |  |
| Do you know if your nursing home has an ACP policy? (*yes/no)* | X | X | X |  |
| 7. Did you ever received information about ACP? |  |  | X | X |
| 8. Did you talk to a resident in this nursing home about the following, in the last 6 months? *(multiple choice: future care and related wishes/ preferences, death and dying or advance directives)* |  |  | X | X |
| 9. Did you talk to a resident in this nursing home about the following, in the last 6 months? *(multiple choice: future care and related wishes/ preferences, death and dying or advance directives)* |  |  | X | X |
| 10. Do you sometimes function as an intermediary between resident and healthcare professional (e.g. signaling wishes from resident to healthcare professional) |  |  | X | X |
| 10. Do you sometimes function as an intermediary between resident’s family and healthcare professional |  |  | X | X |
